# Dropped Gallstones Causing a Perihepatic Abscess and Empyema

**DOI:** 10.1155/2015/629704

**Published:** 2015-06-02

**Authors:** J. R. Robinson, J. K. Wright, S. K. Geevarghese

**Affiliations:** Department of General Surgery, Vanderbilt University Medical Center, Nashville, TN 37232, USA

## Abstract

Iatrogenic perforation of the gallbladder during laparoscopic cholecystectomy is a well-known occurrence; however, the consequences of spillage of gallstones in the peritoneum and particularly intrathoracic complications are less defined. We describe the delayed development of a perihepatic abscess and empyema in a patient five years following laparoscopic cholecystectomy secondary to dropped gallstones. A 53-year-old man with medical history significant for a laparoscopic cholecystectomy five years prior to acute cholecystitis presented with purulent cough, hemoptysis, night sweats, and right-upper quadrant (RUQ) pain. Computed tomography (CT) scan revealed 5.4 cm right-sided subpulmonic and 5.9 cm perihepatic fluid collections with an 8 mm focal radiopaque density within the perihepatic fluid collection. Open intra-abdominal exploration resulted in retrieval of a 1 cm intraperitoneal gallstone. Laparoscopic cholecystectomy is a common surgical operation during which gallstone spillage can occur, causing both intra-abdominal and intrathoracic complications, presenting even years after surgery. This necessitates an attempt to retrieve all free intra-abdominal gallstones during the initial operation.

## 1. Background

Laparoscopic cholecystectomy is a commonly performed and well-established minimally invasive operation; however, there remain known adverse events and potential complications. Iatrogenic perforation of the gallbladder during laparoscopic cholecystectomy is a recognized occurrence with many case reports of complications secondary to dropped stones. This is in contrast to the reported incidence of dropped gallstones after open cholecystectomy, which is extremely rare [1]. Laparoscopically, surgeons may attempt extraction of dropped stones; however, stones may become fragmented, inaccessible, or overlooked, leaving gallstones within the intraperitoneal cavity. There is a reported incidence of gallbladder perforation of 10–40% [2–5]. In a retrospective study of 3686 patients who underwent laparoscopic cholecystectomy, gallstone spillage occurred in 254 patients or 6.9% [3]. Unretrieved dropped stones occur in 1% to 2.4% of total laparoscopic cholecystectomies [2, 3].

Reported complications after gallbladder perforation and gallstone spillage are low; however, the likelihood of a complication increases when gallstones are not retrieved [2]. Although postoperative abscess formation is usually within the first year following cholecystectomy, postoperative residual gallstones have been shown to contribute to delayed abscess formation up to 15 years following cholecystectomy [6]. Thoracic complications specifically are rarely reported.

In this paper we describe a case report of delayed development of a perihepatic abscess and empyema five years following laparoscopic cholecystectomy as a result of dropped gallstones and provide a review of the literature on dropped gallstones during cholecystectomy.

## 2. Case Description

**Figure 1:**
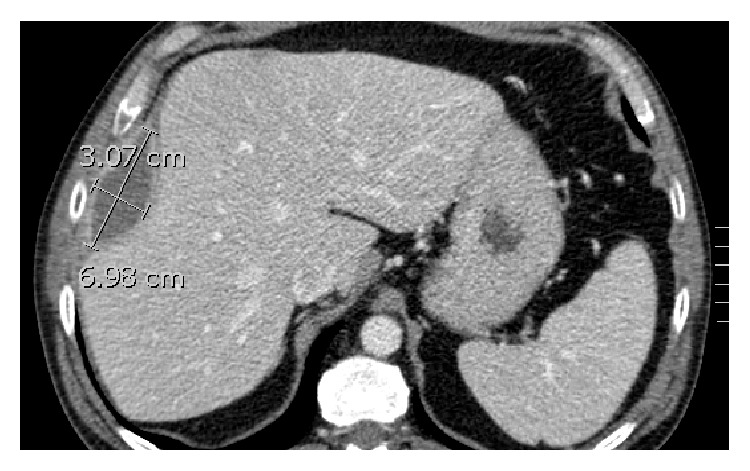
Gallstone-associated perihepatic abscess on preoperative computed tomography measuring 6.98 cm × 3.07 cm.

**Figure 2:**
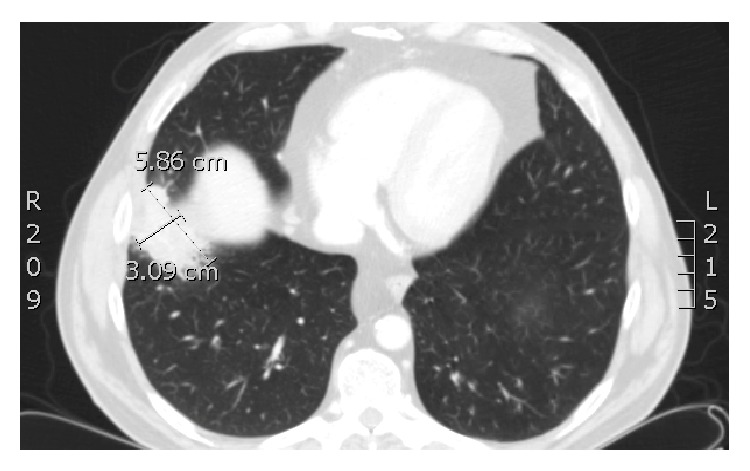
Gallstone-associated subpulmonic fluid collection on preoperative computed tomography measuring 3.09 cm × 5.86 cm.

**Figure 3:**
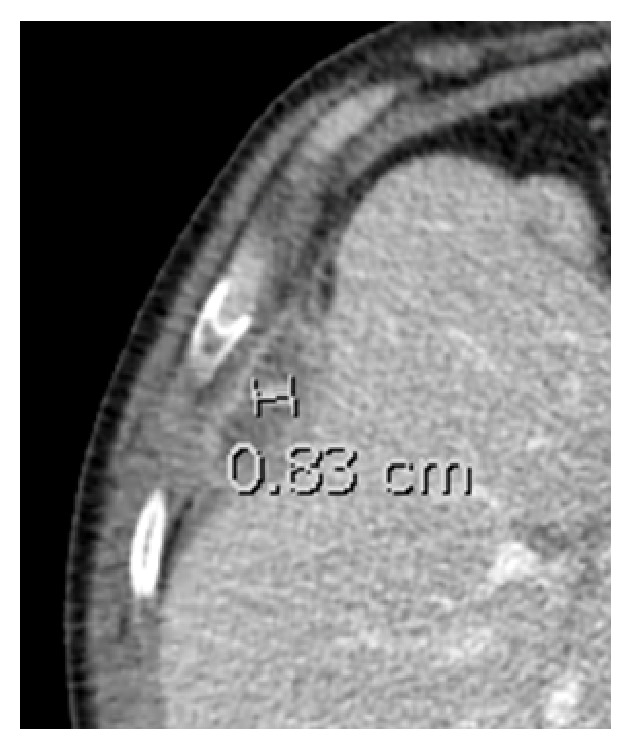
Retained gallstone 5 years following laparoscopic cholecystectomy visualized with computed tomography with a 1 cm radiopaque density within perihepatic fluid collection.

A 53-year-old male presented with approximately 2 months of purulent cough, hemoptysis, night sweats, and RUQ pain. Past medical history consisted of diabetes mellitus, hypertension, and laparoscopic cholecystectomy for acute cholecystitis five years earlier. Per operative report, the laparoscopic cholecystectomy was uncomplicated without mention of gallbladder perforation or gallstone spillage. On physical examination the patient had normal vital signs. He reported pain to palpation in the right-upper quadrant of the abdomen. All laboratory values were within normal limits. A CT scan of the chest, abdomen, and pelvis with intravenous contrast revealed a 5.9 cm perihepatic fluid collection (Figure 1) and 5.4 cm right-sided subpulmonic fluid collection (Figure 2). In addition, an 8 mm focal radiopaque density was visualized within the perihepatic fluid collection (Figure 3).

The diagnosis of intra-abdominal abscess and associated empyema was suspected; therefore, the patient underwent a diagnostic laparoscopy for confirmation. Upon laparoscopy, dense perihepatic adhesions were discovered and conversion to an open intra-abdominal exploration via a subcostal incision was performed. A fistulous tract through the diaphragm was palpated. All adhesions were lysed and the subphrenic abscess cavity was opened and drained. A 1 cm gallstone was retrieved. A drain was left in place. Intraoperative cultures grew pansensitive* Klebsiella*. The patient was placed on a short course of antibiotics and the drain was removed on postoperative day 1.

The patient's symptoms greatly improved during the initial postoperative period and both the perihepatic and subpulmonic fluid collections decreased in size on a follow-up CT scan. Two months following the operation, the patient presented again with hemoptysis. CT-guided drain placement was performed in a residual perihepatic fluid collection. Four weeks of oral antibiotics was prescribed for* Klebsiella* and* E. coli* discovered in drain cultures. There were no further complications and the patient's symptoms are improved.

## 3. Discussion

Laparoscopic cholecystectomy is a common surgical operation during which gallstone spillage can occur, with an incidence exceeding that of the open surgical method. The incidence of gallbladder perforation ranges in the literature with a reported rate of 10% to 40% and retained stones in 1%–13% of laparoscopic cholecystectomies. There are three maneuvers during laparoscopic cholecystectomy reported as being associated with gallbladder perforation. First, the gallbladder is retracted to assist in obtaining the critical view in the dissection of the cystic duct and cystic artery. Secondly, the gallbladder may be perforated during separation of the gallbladder from the liver bed. Lastly, the gallbladder is generally extracted from the abdomen via a small incision and high pressure can result in gallbladder perforation [7].

Despite a high rate of gallbladder perforation and gallstone spillage, the full implication of retained gallstones is unclear. Unretrieved gallstones within the peritoneal cavity may act as a nidus for future infectious complications, such as in this case of intra-abdominal abscess leading to a diaphragmatic fistulous tract and empyema. This tract is thought to have allowed for transdiaphragmatic migration of fluid and bacteria into his thoracic cavity, leading to late empyema formation. There is minimal literature on thoracic complications secondary to retained gallstones following laparoscopic cholecystectomy [8, 9]. Although extremely rare, in patients with persistent pulmonary complaints, evidence of radiolucent foreign body, and a history of laparoscopic cholecystectomy, consideration should be made for potential of retained gallstones serving as the nidus for continued infection.

In contrast to laparoscopic cholecystectomy during which gallstone spillage is an acknowledged potential event, few reports of dropped gallstone complications have been reported after open cholecystectomy [1]. Therefore, some previous individuals have proposed conversion to open cholecystectomy in the case of gallbladder perforation and stone spillage. In a retrospective review of 547 patients with dropped gallstones intraoperatively without a conversion to open procedure for stone removal, only 8 patients (0.08%) developed a postoperative abscess requiring reoperation [10]. Due to this low incidence of complications, we do not recommend conversion to open surgery solely for gallstone retrieval. Rather we recommend meticulous attempts to retrieve gallstones laparoscopically.

Potential intra-abdominal and intrathoracic significant complications of dropped gallstones can occur. This necessitates precautions against gallbladder rupture and stone spillage. Although not an indication for conversion to an open procedure, if gallstone spillage occurs, a reasonable effort should be made in attempt to retrieve all free intra-abdominal gallstones during the initial operation.
